# Cyberviolence among young university students’ intimate partners: an analysis in light of gender and generation categories

**DOI:** 10.1590/0034-7167-2025-0395

**Published:** 2026-09-28

**Authors:** Thammy Novakovski, Rafaela Gessner Lourenço, Tatiane Herreira Trigueiro, Danyelle Leonette Araújo dos Santos, Aida Maris Peres, Bruna da Costa Bueno

**Affiliations:** I Universidade Federal do Paraná. Curitiba, Paraná, Brazil; II Universidade Federal do Rio Grande do Norte. Natal, Rio Grande do Norte, Brazil

**Keywords:** Intimate Partner Violence, Gender-Based Violence, Cyberbullying, Gender Studies, Young Adult., Violencia de Pareja, Violencia de Género, Ciberacoso, Estudios de Género, Adulto Joven.

## Abstract

**Objectives:**

to analyze young university students’ perceptions and experiences regarding cyberviolence between intimate partners and its connections to gender and generational issues.

**Methods:**

an exploratory study with a qualitative approach, grounded in the Theory of Praxis Intervention in Collective Health Nursing and the gender and generation analytical categories. Twenty-nine university students were interviewed between April and June 2024. Content analysis was conducted using MaxQDA version 24.2.0.

**Results:**

cyberviolence among intimate partners primarily affects young women and reproduces the same power imbalances found in offline contexts in the digital environment. Its manifestations include exposure, control, and threats, intensifying during breakups and generating repercussions on health, social relationships, and academic performance.

**Final Considerations:**

cyberviolence, intersected by gender and generational inequalities, makes women vulnerable from a young age and demands critical approaches that guide coping strategies based on equity and social justice.

## RESUMEN

**Objetivos:**

analizar las percepciones y experiencias de jóvenes universitarios respecto a la ciberviolencia en la pareja y su relación con cuestiones de género y generacionales.

**Métodos:**

estudio exploratorio con enfoque cualitativo, basado en la Teoría de la Intervención Praxis en Enfermería de Salud Colectiva y las categorías analíticas de género y generación. Se entrevistó a 29 estudiantes universitarios entre abril y junio de 2024. El análisis de contenido se realizó con el *software* MaxQDA, versión 24.2.0.

**Resultados:**

la ciberviolencia en la pareja afecta principalmente a mujeres jóvenes y reproduce en el entorno digital los mismos desequilibrios de poder que se observan en contextos *offline*. Sus manifestaciones incluyen exposición, control y amenazas, que se intensifican durante las rupturas y generan repercusiones en la salud, las relaciones sociales y el rendimiento académico.

**Consideraciones Finales:**

la ciberviolencia, intersectada por desigualdades de género y generacionales, hace vulnerables a las mujeres desde una edad temprana y exige enfoques críticos que orienten estrategias de afrontamiento basadas en la equidad y la justicia social.

## INTRODUCTION

Cyberviolence is any violent act committed, facilitated, or amplified through the use of information and communication technologies or digital media. This form of violence can aim at control, exposure, harassment, coercion, pressure, monitoring, silencing, threats, and persecution^(1)^. Unlike offline violence, cyberviolence creates a sense of inescapability, as it can occur at any time and in any context^(2)^.

Considering intimate relationships, the online environment has been a fertile ground for the onset of intimate partner violence (IPV), particularly among young people who, due to their generational nature, use technology as an important tool for social interaction. However, young people with an idealized view of affective and sexual relationships may interpret abusive behaviors in the digital space from a romantic perspective^(3)^.

In university settings, predominantly frequented by young people, cyberviolence manifests itself by reflecting and reproducing the dynamics of society. Studies demonstrate a high incidence of this phenomenon among intimate partners in university in countries such as Portugal, Jordan, Mexico, the United States, and Spain^(4-7)^. In an academic context, this violence can lead to dropping out of studies and trigger disorders such as insomnia, mental suffering, substance abuse, and suicide^(8)^.

Research on cyberviolence among university students focuses primarily on its prevalence, associated factors, and social, academic, and physical and mental health consequences for victims and perpetrators^(9,10)^. However, there is a gap in these investigations, as they do not analyze the results in light of gender and generation categories, which are fundamental to the historical and social understanding of the phenomenon, given that cyberviolence primarily affects young women^(7,11)^.

Given this, this study seeks to answer the following question: what are the young university students’ perceptions and experiences regarding cyberviolence between intimate partners, and how do these relate to gender and generational issues?

## OBJECTIVES

To analyze young university students’ perceptions and experiences regarding cyberviolence between intimate partners and its connections to gender and generational issues.

## METHODS

### Ethical aspects

This study is part of a master’s thesis entitled “*Ciberviolência entre parceiros íntimos universitários: uma análise à luz das categorias gênero e geração*”, developed in the Graduate Program in Nursing at the *Universidade Federal do Paraná*. Conducted in accordance with Resolution 466/2012 of the Brazilian National Health Council, it was approved by the Research Ethics Committee of the Health Sciences Sector of the same institution. All participants signed the Informed Consent Form. To guarantee anonymity, the discourses were coded with the letter “E”, followed by the interview number (for instance, “E1” for the first).

### Theoretical-methodological framework

The gender and generation analytical categories were adopted as the theoretical-philosophical framework. The gender category is a category of historical analysis of power relations sustained and constituted from the naturalization of social characteristics attributed to each gender. The generation category explores the relationships between people of the same age group and how they relate to the historical context.

The theoretical and methodological framework used in this study was the Theory of Praxis Intervention in Collective Health Nursing (TPICHN). This theory focuses on research and intervention in nursing within the community’s health-disease process. This study concentrated on the first two stages of TPICHN, namely: 1) capturing objective reality; 2) interpreting objective reality^(12)^.

### Study design

This is an exploratory and descriptive study with a qualitative approach. The criteria established for the COnsolidated criteria for REporting Qualitative studies were observed in the research design and development.

### Study setting

The study was developed at a public university located in the city of Curitiba, Paraná, specifically in the institution’s Health Sciences Sector. This sector was chosen because of its relevance in training professionals capable of addressing social challenges, such as violence, and contributing to promoting health and well-being in society.

### Population

Young people aged 18 to 29, regularly enrolled in undergraduate courses in the Health Sciences Sector, were included. This sector comprises six courses, namely medicine, dentistry, nutrition, nursing, pharmacy, and occupational therapy. No exclusion criteria were established.

### Data collection

The research was disseminated through institutional emails, with the support of academic centers and course coordinators, as well as through posters in student areas. Those interested filled out an electronic form, received information about the study objectives and, with their consent, were contacted to schedule the interviews.

Data collection took place between April 24 and June 4, 2024, through in-depth interviews, preceded by two pilot interviews aimed at validating the instrument. The interviews were conducted in person, in a private setting, by appointment according to participants’ availability. The interviews were conducted by a nurse and researcher experienced in the subject matter, with no prior relationship to the interviewees.

The interview script initially included characterizing participants through questions about age, sex, race, marital status, marital status, education, occupation, family income, religion, number of children, course of study, and relationship status. Following this, open-ended questions were addressed to explore knowledge and perceptions about cyberbullying by intimate partners, its causes, repercussions, and coping strategies, as well as personal or third-party experiences.

The following were investigated for participants who reported experiencing cyberviolence: the types of violence suffered; the relationship and gender of the perpetrator; the emotional and health impacts; the search for support from health services and the university; the effectiveness of these services; the filing of complaints; and the perceived needs. Finally, the role of the university in addressing cyberviolence was explored based on participants’ expectations and suggestions. All interviews were recorded on a digital voice recorder, totaling six hours, 56 minutes, and 39 seconds of audio.

To reduce bias, the concept of cyberviolence was introduced via rapport. The interviews were recorded to ensure data integrity and preserve anonymity, and a field diary was used to record impressions and challenges. In total, 42 people expressed interest, of whom 29 participated; the others withdrew due to unavailability, interruption of contact, or non-attendance.

### Data analysis

Data were analyzed using content analysis^(13)^. Content analysis was divided into three stages: 1) pre-analysis; 2) material exploration; 3) processing of results and interpretation^(13)^.

The Max Qualitative Data Analysis (MaxQDA^®^) version 24.2.0 was used to organize and analyze qualitative data, allowing for editing, visualization, and linking of documents with complete control by the researcher. MaxQDA^®^ was used via the direct coding function, automatically assigning codes to the data and organizing them into code trees in the internal source system. Quantitative data were organized in a spreadsheet and subjected to descriptive analysis, presented using absolute frequencies.

## RESULTS

Twenty-nine young university students, aged between 18 and 29 years, participated in the study. The majority were female (n=25) and four were male. Participants were students of nursing (n=6), medicine (n=6), occupational therapy (n=5), pharmacy (n=5), nutrition (n=4), and dentistry (n=3).

In relation to skin color, 21 identified as white, three as black, three as mixed-race, one as indigenous, and one as Asian. Concerning religion, the majority identified as Christian (n=16), while one identified as Umbanda, another as pagan, and 11 did not follow a specific religion. As for sexual orientation, heterosexual individuals predominated (n=18), followed by bisexual (n=7), homosexual (n=2), pansexual (n=1), and demisexual (n=1) individuals. In terms of marital status, 28 were single, of whom 17 were in a romantic relationship, and one was married.

Regarding knowledge of people who have experienced cyberviolence, two-thirds of respondents (n=21) stated that they knew someone who had gone through this experience. Of these, about half (n=10) mentioned colleagues or acquaintances; nine indicated friends; and two mentioned family members.

The most frequently mentioned form was image-based sexual abuse (IBSA), defined as the creation, distribution, or threat of sharing intimate content without consent, reported in 16 accounts. Threats were identified in four cases. Other manifestations of cyberviolence were less frequent and included controlling behaviors, harassment, gaslighting-characterized by psychological manipulation aimed at undermining the victim’s perception of reality-virtual rape-which involves coercion into non-consensual sexual practices in digital environments-and manipulation, each of which was reported in only one account.

Concerning personal experiences, almost two-thirds of participants (n=19) reported having suffered cyberviolence, with 12 of them involving intimate partners. The most common forms were threats and control, recorded in six reports, followed by exposure in four cases, IBSA and gaslighting in three, while stalking and virtual rape appeared in one case each.

From data analysis, four categories emerged: Perceptions of cyberviolence; Experiencing cyberviolence; Consequences of cyberviolence; Feelings towards cyberviolence.

The “Perceptions of cyberviolence” category describes how cyberviolence can be a tool to exert control over others, reflecting the dynamics of gender domination identified in society. Participants pointed out that the digital environment amplifies these abusive practices, with sexism being one of the main pillars of this phenomenon.

*Look, it’s more because of* [...] *because of this idea that men have dominance over us, you know?* (E1)

*The reasons I believe that encourage these acts of violence are revenge, for instance, when a man feels very wronged by his partner, and he knows that she has a social disadvantage online, and so he finds different ways to get revenge on her.* (E28)

The reports highlighted how cyberviolence begins early in individuals’ lives, with cases of IBSA and online harassment being common during adolescence. Reports of IBSA during this period refer exclusively to women who were exposed.

*Ah, back when I was an adolescent, at my school, there were a lot of cases of girls’ photos and videos being leaked. Quite a few* [...]. (E20)

*I had two friends. I remember one of them* [...] *she was always dating other guys and she had nude photos leaked. We were in sixth grade.* (E19)

*I knew her from elementary school. She had already sent an intimate photo to her boyfriend. Another friend of that boyfriend saw it and spread it all over the school. We were under 14 years old.* (E22)

The “Experiencing cyberviolence” category reveals participants’ perception that cyberviolence is common, particularly in romantic relationships. The reports showed that threats of exposing intimate photos were used to control and blackmail victims, especially during or after the end of a relationship, in order to force the relationship to continue or to silence the victim to preserve the abuser’s reputation. Furthermore, it was evident that abuse can persist through online stalking, with the creation of fake profiles for defamation and emotional manipulation, such as gaslighting.

*I dated him for two years, and these threats happened during the relationship, especially when I wanted to break up* [...]*. And, after I broke up, to see if I would get back together and also to prevent me from saying anything he might have done to me, or confiding in someone so as not to tarnish his reputation.* (E25)

*He’s still trying to send* [friend requests on social media]. [...] *so, all my social media reports are private because he even made a fake account to control me.* (E6)

The social construction of romantic love influences the perception of cyberviolence, normalizing it and making abuse difficult to recognize. In this regard, reports have shown that control and emotional manipulation begin subtly, disguised as acts of care, highlighting the romanticization of possessiveness.

*And when you’re the one suffering through these things, I think it happens so gradually that you don’t even realize it. You don’t notice. It’s just another day, another action, he likes me so much. He’s worried about me. And also, the forms of manipulation end up becoming so profound that you fall even more in love.* (E21)

*He* [...] *started saying that he couldn’t live without me, that he couldn’t be away from me for very long. Then there’s that thing where at first you think it’s cute. You think, “Wow, he can’t live without me, how sad”.* (E1)

The “Feelings towards cyberviolence” category encompasses anger, shame, fear, and self-devaluation. The reports highlighted shame as a feeling that led to the silencing of violent situations for fear that the suffering would be considered insignificant or discredited. The feeling of fear was related to the possibility of public exposure of intimate images and the social and emotional consequences of cyberviolence.

*I didn’t tell anyone because you get embarrassed, you know? (*E29)

[...] *even though I’m very far from the place and haven’t seen him in six years, I’m still afraid.* (E25)

The feeling of discomfort was evident in the report of one of the participants, who stated that he had been pressured by his girlfriend to participate in online sexual activities, having given in to the pressure because he believed that, as a man, he should not show vulnerability. Furthermore, he reported that he gave in out of fear of negative consequences and the perception that a complaint about violence suffered by a man would not be taken seriously.

*I ended up letting things happen* [sexual acts] *because I was afraid there might be bad repercussions if I didn’t. Or because I thought that as the guy in the relationship, I shouldn’t be the one feeling uncomfortable.* (E18)

The “Consequences of cyberviolence” category revealed the impacts of this form of violence on victims’ health and daily lives. The reports showed feelings of invalidation and guilt that led to questioning their own worth and the legitimacy of their reactions. Notably, one of the interviewees stated that she felt forced to move to another state, facing consequences such as isolation, prejudice, and a worsening of her emotional state.

*For a long time, it messed with my self-esteem.* [...] *they* [my ex-boyfriend’s friends] *said things about my appearance, that I was crazy, and so on. I think it affected me for a long time because I started to think that maybe they were right about the things I complained about* [...]. (E9)

*I had to leave.* [...] *I think the consequences still haunt me today* [...] *and how depressed I became being here, far from where I lived and where I was from.* [...] *here I suffered various racial and ethnic issues. I think it was a domino effect, from cyberviolence to me arriving here.* (E25)

Participants pointed out that people experiencing cyber violence may face panic attacks, anxiety, depression, and in some cases, attempt suicide as a result of the emotional distress caused by cyber violence.

*I started having panic attacks. I didn’t know what a panic attack was.* [...] *and one day, I had a very strong panic attack, and then I was hospitalized because I had them every day and I thought I was going to die.* (E25)

[...] *I once had a case where a friend of mine felt so humiliated and depressed that she tried to take her own life because her family found out.* (E6)

Participants stated that isolation, lack of concentration, and emotional distress due to cyberviolence frequently lead to negative consequences for academic life, such as absences from classes, failing grades, and delays in the academic schedule. This is due to the fragility of their psychological state, which impairs university performance, making it difficult to keep up with classes and take exams.

*Right after he did what he said he would* [share intimate images without consent]*, I isolated myself quite a bit.* [...] *so, I stopped going to school.* [...] *I stopped going, I fell behind a year. And it was really hard for me to go back to school again.* (E25)

*I did poorly on a test, I missed a class, and then, when the threats started and he began to stalk me, I would sometimes get nervous when I was at college, more distracted than usual.* (E6)

## DISCUSSION

The forms of cyberviolence between intimate partners most experienced by participants were threats and control, followed by non-consensual exposure of intimate information and IBSA, usually accompanied by manipulation and coercion. Among the cases mentioned involving third parties, IBSA was the most cited, possibly reflecting the common misconception that cyberviolence among intimate partners is limited to the dissemination of intimate images.

However, this simplified perception does not encompass the complexity of the experiences of those who suffer this type of violence. Behaviors such as excessive control, threats, emotional manipulation, and gaslighting are recurrent in abusive relationships, but sometimes remain invisible until culminating in explicit episodes, such as IBSA^(14)^. In general, these experiences are not shared with the support network, being recognized only when they materialize in public displays or face-to-face violence. Thus, it can be inferred that cyberviolence between intimate partners encompasses a broad and invisible set of abusive practices that go beyond cases of exposure. Furthermore, cyberviolence can be the first indication of abuse in intimate relationships and manifest itself in conjunction with other forms of violence, an aspect already evidenced in previous studies^(15,16)^.

The “Perceptions of cyberviolence” category reveals that participants perceive violence in the digital environment as a means of punishment or revenge used by men against women, exploiting their social disadvantage in this space. The experiences of women facing cyberviolence differ from those of men, as they are marked by gender inequalities, reinforcing the need to analyze these issues from a perspective that considers this inequality^(11)^.

Cyberviolence as a form of gender-based violence was identified in research conducted with young Arabs, which found its manifestation through coercion and power imbalances between men and women in a patriarchal society. In this society, men are encouraged to display aggression, adopt controlling behaviors, and promote gender discrimination based on stereotypes. Meanwhile, women are taught to accept and submit to men, exposing them to exploitation and abuse, both in the real world and in the virtual environment^(5)^.

Another example of cyberviolence as gender-based violence was detected in a study that investigated discourses and practices of violence against girls and women in anonymous internet forums: the chans. The study analyzed 9.5 million posts in ten chans and found that, in 46% of discussions about women in these forums, violent terms were used to refer to them, a percentage that rises to 69% when conversations are related to pornography. In these spaces, women are seen as targets of resentment, hatred, and objectification^(17)^.

Although the internet may seem like a space for neutralizing gender differences, it is observed that it reproduces and intensifies gendered stereotypes present in society. Thus, cyberviolence emerges as an instrument for reaffirming hegemonic masculinity, transposing the same power asymmetries observed in the offline context to the digital environment^(5,18)^.

The results of this study indicate that cyberviolence manifests itself early in women’s lives and is often normalized, making it rare to reach adulthood without experiencing abuse. Therefore, an analysis focused exclusively on the perspective of gender vulnerability would not be comprehensive enough to understand the phenomenon, and it is also necessary to analyze it from a generational perspective.

Some studies link IBSA to adolescent recklessness or impulsivity, reinforcing victim-blaming^(19,20)^. Others, whose focus is neurological development, establish an association between the sending of erotic media and a lack of awareness or underdevelopment of the prefrontal cortex in adolescents^(15,21)^. However, these approaches ignore the fact that such practices also occur in a planned manner among adults, disregarding the structural complexity of cyberviolence^(16)^.

The idea of recklessness can also be seen in the narratives of participants who experienced cyberviolence when they blame themselves for the violence suffered, interpreting these experiences as the result of behaviors considered inappropriate, naive, or impulsive, seen as typical of adolescence. In this way, they reproduce the logic of victim-blaming, perpetuating the stigmas that surround them^(22,23)^.

Therefore, the analysis of the results of this study, from a generational perspective as part of TPICHN’s interpretation of objective reality, shows that the vulnerability of young people to cyberviolence is accentuated by the growing and increasingly early insertion into the digital environment as a place for initiating and maintaining affective and sexual relationships^(3,22)^. In this context, tackling cyberviolence requires including digital interactions in prevention strategies, due to their impact on the lives of girls and women.

The “Experiencing cyberviolence” category indicates that this form of violence is common in romantic relationships, mainly experienced through threats and control. Supporting these results, a qualitative study revealed that control is a common denominator in experiences of cyberviolence by an intimate partner and that, even after the breakup, there was an increase in unwanted contact with the ex-partner, who attempted to maintain influence over the woman. To this end, abusers used various technologies, such as location tracking and account hacking, resulting in invasions of privacy, constant calls, and threatening messages^(14)^.

The normalization of controlling behaviors is based on the patriarchal discourse that they are demonstrations of love and care. This social construct, which associates romantic love with possession, plays a central role in the perception of cyberviolence, as it hinders the recognition of abusive behaviors and legitimizes abusive attitudes and digital surveillance within the relationship. In doing so, it reinforces emotional dependence and perpetuates the cycle of violence^(3)^. Overcoming these dynamics requires a social transformation capable of questioning and redefining romantic love, challenging the cultural norms that link control and possessiveness to care.

The “Feelings towards cyberviolence” category highlights the negative emotions triggered by experiencing cyberviolence, particularly shame. This feeling often leads victims to remain silent and avoid seeking help, fearing that their experiences will be minimized, especially in relationship contexts.

Shame, common in situations of violence against women, intensifies in cases of unauthorized exposure of intimacy, functioning as a mechanism that perpetuates violence and hinders reporting. It is important to note that shame is the result of an internalization process in which the victim adopts negative self-assessments. Culturally, honor is associated with men, while shame is attributed to women, reinforcing a hierarchy that devalues the female image^(24)^.

Hence, shame can keep individuals in situations of violence, as evidenced in the statement of a male interviewee who, despite discomfort, reported having participated in online sexual activities due to pressure from his girlfriend. This experience can be analyzed from a patriarchal perspective, which views virility as a socially constructed ideal, simultaneously representing a privilege and a trap. Thus, virility-associated with strength and reproductive capacity-imposes on men the constant obligation to prove their masculinity, making them vulnerable to situations of oppression^(25,26)^. In this context, the pressure to conform to gender expectations can hinder the recognition and reporting of abusive experiences lived by men, perpetuating dynamics of violence that are not made visible.

The “Consequences of cyberviolence” category highlights that the impacts of this form of violence extend beyond the online space and affect the daily lives of victims, including self-esteem, confidence, and social interactions. People experiencing cyberviolence frequently face feelings of invalidation, guilt, and fear, which can result in significant changes in their routine, such as avoiding certain places or even moving to another city^(27)^.

These social impacts are supported by authors who use the expression “social death” to describe reports of moral destruction in the eyes of family, friends, and strangers on the internet experienced by people in situations of cyberviolence. Furthermore, there is a feeling of persecution and insecurity that makes the person feel constantly watched and judged, regardless of where they are, even if no one in that location has access to the violated content^(27)^.

The harms of cyberviolence are felt at both individual and systemic levels, affecting individuals’ privacy, autonomy, and psychological well-being^(11)^. Regarding mental health, cyberviolence can trigger depression, panic attacks, anxiety, and, in more severe cases, suicide attempts. A study conducted with 970 adolescents from sexual minorities, aged between 14 and 17, investigated the relationship between IBSA and suicidal and self-harming behaviors, finding that the threat of disseminating this type of content was associated with an increased risk of suicidal ideation, planning and suicide attempts, as well as self-harm. Non-consensual posting of media was also linked to a higher risk of suicidal ideation and attempts^(28)^.

Despite numerous studies pointing to the negative impacts of cyberviolence on health^(2,5,9,11,19,28)^, the health sector still does not recognize it as an object of its intervention. Furthermore, there is a lack of Brazilian studies on this topic, which may be related to the low visibility of the phenomenon in the country and the consequent absence of effective measures for its prevention and control.

Therefore, it is necessary for universities, as privileged spaces for promoting health, to incorporate the fight against violence, including its digital manifestations, as a central axis of their institutional policies. Experiences such as the “General Guidelines for Institutional Intervention Actions in Situations of Gender and Sexual Orientation Violence and Discrimination” from the *Universidade de São Paulo* illustrate relevant initiatives by establishing normative parameters, support services, and protocols for addressing gender-based violence, reaffirming the commitment of higher education institutions to building safe, respectful academic environments aligned with the Sustainable Development Goals^(29)^.

It is noteworthy that the exclusive inclusion of students from the health field as participants in the study fosters critical reflection on inequalities and structures that perpetuate violence. This process can improve future care practices by promoting the recognition of these professionals as agents of change capable of overcoming limitations imposed by historical and cultural processes of dependence and subordination, enabling the construction of practices grounded in equity, rights, citizenship, and social justice^(23)^.

### Study limitations

A key limitation of this study is the low participation of men, even with frequent invitations. Men’s reluctance to address violence, whether as victims or perpetrators, contributed to the research predominantly reflecting female experiences. However, this raises questions about male silence surrounding the issue of IPV, including the invisibility of the violence they also experience in intimate relationships.

### Contributions to nursing, health, or public policy

This study analyzes cyberviolence from a critical perspective, recognizing it as an emerging phenomenon that is still incipiently addressed in the fields of health and nursing. It is a contemporary expression of gender violence, structured by power asymmetries derived from sociocultural constructions of gender and generation. By highlighting its impacts on young people’s health-disease process, especially women, this investigation broadens the understanding of the symbolic and relational dynamics that underpin practices of control and domination mediated by digital technologies.

Given the scarcity of national studies exploring this issue from an intersectional perspective, the findings of this work reaffirm the role of nursing as an ethical, critical, and transformative practice. This research contributes to consolidating cyberviolence as an object of attention in the fields of health surveillance, continuing education, and interprofessional care, especially in university settings. Furthermore, it provides support for the development of intersectoral strategies and interventions committed to promoting health, equity, and building safe, welcoming, and inclusive environments.

## FINAL CONSIDERATIONS

The findings of this study show that cyberviolence is a frequent reality among young university students, especially women, beginning in adolescence and occurring more frequently in the context of intimate relationships, particularly during breakups. Its manifestations, such as control, coercion, harassment, exposure of intimate images, and stalking, have significant impacts on physical and mental health, social relationships, and academic performance.

Cyberviolence reveals itself as an expression of symbolic violence sustained by structural gender and generational inequalities, which reinforce practices that humiliate, dehumanize, and make vulnerable primarily young women. It is important to highlight that the theoretical and methodological framework adopted in this study recognizes that a perspective sensitive to other social categories, such as social class and race/ethnicity, is essential for rethinking power relations in the dynamics of cyberviolence. In the fields of public health and nursing, this approach broadens the understanding of the impacts of cyberviolence on the health-disease process, associating them with structural inequalities.

Therefore, overcoming cyberviolence requires transforming the power relations that sustain it, which is more important than punitive responses. Thus, this study contributes to this debate, encouraging new research and interventions in the university context that promote equity, respect, and social justice.

## Data Availability

The research data are available within the article.
