# Supplementary material for: Cyberviolence among young university students’ intimate partners: an analysis in light of gender and generation categories
Source: Rev Bras Enferm. 2026 Sep 28;79:e20250395. doi: 10.1590/0034-7167-2025-0395 (PMC13623388; doi:10.1590/0034-7167-2025-0395)
Supplement: Translation

# Translation: Ciberviolência entre parceiros íntimos jovens universitários: análise à luz das categorias gênero e geração

**DOI:** 10.1590/0034-7167-2025-0395pt

**Authors:** Thammy Novakovski, Rafaela Gessner Lourenço, Tatiane Herreira Trigueiro, Danyelle Leonette Araújo dos Santos, Aida Maris Peres, Bruna da Costa Bueno

**Affiliations:** I Universidade Federal do Paraná. Curitiba, Paraná, Brasil; II Universidade Federal do Rio Grande do Norte. Natal, Rio Grande do Norte, Brasil

**Keywords:** Violência por Parceiro Íntimo, Violência de Gênero, Vitimização Cibernética, Estudos de Gênero, Adulto Jovem.

## Abstract

**Objetivos:**

analisar as percepções e vivências de jovens universitários sobre a ciberviolência entre parceiros íntimos e suas articulações com as questões de gênero e geração.

**Métodos:**

estudo exploratório, de abordagem qualitativa, fundamentado na Teoria da Intervenção Práxica da Enfermagem em Saúde Coletiva e nas categorias analíticas gênero e geração. Foram entrevistados 29 universitários entre abril e junho de 2024. A análise de conteúdo foi conduzida com apoio do *software* MaxQDA, versão 24.2.0.

**Resultados:**

a ciberviolência entre parceiros íntimos afeta, sobretudo, mulheres jovens, e reproduz no ambiente digital as mesmas assimetrias de poder do contexto *offline*. Suas manifestações incluem exposição, controle e ameaças, intensificando-se durante rompimentos e gerando repercussões na saúde, nas relações sociais e no desempenho acadêmico.

**Considerações Finais:**

a ciberviolência, atravessada por desigualdades de gênero e geração, vulnerabiliza mulheres desde a juventude e demanda abordagens críticas que orientem estratégias de enfrentamento pautadas na equidade e na justiça social.

## INTRODUÇÃO

A ciberviolência é qualquer ato violento cometido, facilitado ou amplificado pelo uso de tecnologias da informação e comunicação ou mídias digitais. Essa forma de violência pode ter como finalidade o controle, a exposição, o assédio, a coerção, a pressão, o monitoramento, o silenciamento, a ameaça e a perseguição^(1)^. Diferentemente da violência *offline*, a ciberviolência cria uma sensação de inescapabilidade, pois pode ocorrer a qualquer momento e contexto^(2)^.

Considerando relacionamentos íntimos, o ambiente *online* tem sido um espaço propício para o início de atitudes de violência entre parceiros íntimos (VPI), com destaque para o público jovem que, por sua natureza geracional, utiliza a tecnologia como importante ferramenta de interação social. Contudo, jovens com uma visão idealizada de relacionamentos afetivos e sexuais podem interpretar comportamentos abusivos no espaço digital por uma perspectiva romântica^(3)^.

No ambiente universitário, majoritariamente frequentado por jovens, a ciberviolência se manifesta refletindo e reproduzindo as dinâmicas da sociedade. Estudos demonstram alta incidência desse fenômeno entre parceiros íntimos universitários em países como Portugal, Jordânia, México, Estados Unidos e Espanha^(4-7)^. No contexto acadêmico, essa violência pode levar ao abandono dos estudos e desencadear transtornos como insônia, sofrimento mental, abuso de substâncias e suicídio^(8)^.

As pesquisas sobre ciberviolência entre universitários focam principalmente na sua prevalência, nos fatores associados e nas consequências sociais, acadêmicas e de saúde física e mental para vítimas e perpetradores^(9,10)^. No entanto, há uma lacuna nessas investigações, pois elas não analisam os resultados à luz das categorias de gênero e geração, que são fundamentais para a compreensão histórica e social do fenômeno, visto a ciberviolência afetar principalmente as mulheres jovens^(7,11)^.

Diante disso, este estudo busca responder à seguinte questão: quais são as percepções e vivências de jovens universitários sobre a ciberviolência entre parceiros íntimos e como elas se relacionam com as questões de gênero e geração?

## OBJETIVOS

Analisar as percepções e vivências de jovens universitários sobre a ciberviolência entre parceiros íntimos e suas articulações com as questões de gênero e geração.

## MÉTODOS

### Aspectos éticos

Este estudo integra a dissertação de mestrado “Ciberviolência entre parceiros íntimos universitários: uma análise à luz das categorias gênero e geração”, desenvolvida no Programa de Pós-Graduação em Enfermagem da Universidade Federal do Paraná. Conduzido conforme a Resolução nº 466/2012 do Conselho Nacional de Saúde, foi aprovado pelo Comitê de Ética em Pesquisa do Setor Ciências da Saúde da mesma instituição. Todos os participantes assinaram o Termo de Consentimento Livre e Esclarecido. Para garantir o anonimato, os discursos foram codificados com a letra “E”, seguida pelo número da entrevista (por exemplo, “E1” para a primeira).

### Referencial teórico-metodológico

Adotaram-se como referencial teórico-filosófico as categorias analíticas gênero e geração. A categoria gênero é uma categoria de análise histórica das relações de poder sustentadas e constituídas a partir da naturalização das características sociais atribuídas a cada gênero. A categoria geração explora as relações entre as pessoas de uma mesma faixa etária e como elas se relacionam com o contexto histórico.

O referencial teórico-metodológico utilizado neste estudo foi a Teoria da Intervenção Práxica da Enfermagem em Saúde Coletiva (TIPESC). A teoria se direciona à pesquisa e à intervenção em enfermagem no processo de saúde-doença da coletividade. Este estudo concentrou-se nas duas primeiras etapas da TIPESC, quais sejam: 1) captação da realidade objetiva; 2) interpretação da realidade objetiva^(12)^.

### Tipo de estudo

Este estudo é exploratório e descritivo, de abordagem qualitativa. Foram observados os critérios estabelecidos para o *COnsolidated criteria for REporting Qualitative studies* na elaboração e no desenvolvimento da pesquisa.

### Cenário do estudo

O estudo foi desenvolvido em uma universidade pública localizada na cidade de Curitiba, Paraná, especificamente no Setor de Ciências da Saúde da instituição. A escolha desse setor foi motivada pela sua relevância na formação de profissionais capacitados para enfrentar desafios sociais, como a violência, contribuindo para a promoção da saúde e do bem-estar da sociedade.

### População

Foram incluídos jovens com idades entre 18 e 29 anos, regularmente matriculados nos cursos de graduação, pertencentes ao Setor de Ciências da Saúde. Esse setor é formado por seis cursos, a saber medicina, odontologia, nutrição, enfermagem, farmácia e terapia ocupacional. Não foram estabelecidos critérios de exclusão.

### Coleta de dados

A pesquisa foi divulgada por e-mails institucionais, com apoio de centros acadêmicos e coordenações de curso, bem como por meio de cartazes em áreas de circulação estudantil. Os interessados preencheram um formulário eletrônico, receberam informações sobre os objetivos do estudo e, mediante consentimento, foram contatados para o agendamento das entrevistas.

A coleta de dados ocorreu entre 24 de abril e 4 de junho de 2024, por meio de entrevistas em profundidade, antecedidas por duas entrevistas piloto destinadas à validação do instrumento. As entrevistas foram realizadas presencialmente, em ambiente reservado, mediante agendamento conforme a disponibilidade dos participantes. A condução foi realizada por uma enfermeira e pesquisadora experiente na temática, sem vínculo prévio com os entrevistados.

O roteiro das entrevistas contemplou, inicialmente, a caracterização dos participantes por meio de perguntas sobre idade, sexo, cor, estado civil, situação marital, escolaridade, ocupação, renda familiar, religião, número de filhos, curso e situação de relacionamento. Em seguida, foram abordadas questões abertas que exploraram o conhecimento e as percepções sobre a ciberviolência por parceiro íntimo, suas causas, repercussões e estratégias de enfrentamento, bem como experiências pessoais ou de terceiros.

Para os participantes que relataram vivência de ciberviolência, investigaram-se os tipos de violência sofridos, a relação e o sexo do autor, os impactos emocionais e à saúde, a busca por apoio em serviços de saúde e na universidade, a efetividade desses atendimentos, a realização de denúncias, e as necessidades percebidas. Por fim, indagou-se o papel da universidade no enfrentamento da ciberviolência a partir das expectativas e sugestões dos participantes. Todas as entrevistas foram registradas em gravador de voz digital, totalizando seis horas, 56 minutos e 39 segundos de áudio.

Para diminuir vieses, o conceito de ciberviolência foi apresentado via *Rapport*. As entrevistas foram gravadas para garantir a integridade dos dados, preservando o anonimato, e foi utilizado um diário de campo para registrar impressões e desafios. Ao todo, 42 pessoas manifestaram interesse, das quais 29 participaram; as demais desistiram por indisponibilidade, interrupção de contato ou não comparecimento.

### Análise dos dados

Os dados foram analisados por meio da análise de conteúdo^(13)^. A análise de conteúdo foi dividida em três etapas, sendo elas: 1) pré-análise; 2) exploração do material; 3) tratamento dos resultados e interpretação^(13)^.

O *software Max Qualitative Data Analysis* (MaxQDA^®^), versão 24.2.0, foi utilizado para organizar e analisar os dados qualitativos, permitindo edição, visualização e interligação de documentos com total controle do pesquisador. O MaxQDA^®^ foi utilizado por meio da função de codificação direta, atribuindo códigos automaticamente aos dados e organizando-os em árvores de códigos no sistema de fontes internas. Os dados quantitativos foram organizados em planilha eletrônica e submetidos à análise descritiva, com apresentação por meio de frequências absolutas.

## RESULTADOS

Participaram da pesquisa 29 jovens universitários, com idades entre 18 e 29 anos, sendo a maioria era do sexo feminino (n=25) e quatro do sexo masculino. Os participantes eram estudantes dos cursos de enfermagem (n=6), medicina (n=6), terapia ocupacional (n=5), farmácia (n=5), nutrição (n=4) e odontologia (n=3).

Quanto à cor de pele, 21 se declararam brancos, três, pretos, três, pardos, um, indígena, e um, amarelo. Quanto à religião, a maioria se identificou como cristã (n=16), enquanto um se declarou umbandista, outro, pagão, e 11 não seguiam religião específica. Quanto à orientação sexual, predominaram heterossexuais (n=18), seguidos por bissexuais (n=7), homossexuais (n=2), pansexual (n=1) e demissexual (n=1). Em termos de estado civil, 28 eram solteiros, dos quais 17 mantinham um relacionamento afetivo, e um era casado.

Sobre o conhecimento de pessoas que vivenciaram ciberviolência, dois terços dos entrevistados (n=21) afirmaram conhecer alguém que passou por essa experiência. Desses, cerca de metade (n=10) mencionou colegas ou conhecidos; nove apontaram amigos; e dois citaram familiares.

O abuso sexual baseado em imagens (Image-Based Sexual Abuse - IBSA), definido pela criação, divulgação ou ameaça de compartilhamento de conteúdos íntimos sem consentimento, constituiu a forma de violência mais prevalente, presente em 16 relatos. As ameaças foram identificadas em quatro casos. Outras manifestações de ciberviolência ocorreram de forma menos frequente, incluindo comportamentos de controle, assédio, gaslighting - caracterizado pela manipulação psicológica destinada a comprometer a percepção da realidade da vítima -, estupro virtual - que envolve a coação para práticas sexuais não consentidas em ambientes digitais -, e manipulação, cada uma mencionada em apenas um relato.

Em relação às experiências pessoais, quase dois terços dos participantes (n=19) relataram ter sofrido ciberviolência, sendo que, entre eles, 12 envolveram parceiros íntimos. As formas mais comuns foram ameaça e controle, registradas em seis relatos, seguidas por exposição, em quatro casos, IBSA e gaslighting, em três, enquanto stalking e estupro virtual apareceram em um caso cada.

Da análise de dados, emergiram quatro categorias, quais sejam: Percepções sobre a ciberviolência; Vivência da ciberviolência; Consequências da ciberviolência; Sentimentos em relação à ciberviolência.

A categoria “Percepções sobre a ciberviolência” descreve como a ciberviolência pode ser uma ferramenta para exercer controle sobre o outro, refletindo as dinâmicas de dominação de gênero identificadas na sociedade. Os participantes apontaram que o ambiente digital potencializa essas práticas abusivas, sendo o machismo um dos principais alicerces desse fenômeno.

*Olha, é mais por causa de* [...] *por uma ideia que homem tem dominação sobre nós, sabe?* (E1)

*Os motivos que eu acredito que incentiva esses atos de violência é vingança, por exemplo, quando o homem se sente muito injustiçado pela parceira, e ele sabe que, na internet, ela tem uma desvantagem social, e aí, nisso, ele encontra diferentes meios de se vingar dela.* (E28)

As falas destacaram como a ciberviolência inicia precocemente na vida dos indivíduos, sendo comuns casos de IBSA e ofensas em ambientes *online* na adolescência. Os relatos de IBSA nesse período se referem exclusivamente a mulheres expostas.

*Ah, nessa época de adolescente, assim, na minha escola, teve muitos casos de vazar foto e vídeos das meninas. Bastante* [...]. (E20)

*Eu tinha duas amigas. Eu lembro que uma delas* [...] *ela sempre ficava com mais meninos e ela teve nude vazado. A gente estava na sexta série.* (E19)

*Eu conhecia ela no fundamental. Ela já tinha mandado uma foto íntima para o namorado dela. Outro amigo desse namorado viu e espalhou para escola inteira. A gente tinha menos de 14 anos.* (E22)

A categoria “Vivência da ciberviolência” revela a percepção dos participantes de que a ciberviolência é comum, particularmente em relacionamentos amorosos. Nos relatos, constatou-se que ameaças de exposição de fotos íntimas eram usadas para controlar e chantagear as vítimas, especialmente durante ou após o término do relacionamento, a fim de forçar a continuidade da relação ou silenciar a vítima para preservar a reputação do agressor. Além disso, evidenciou-se que abuso pode persistir por meio de perseguições *online*, com a criação de perfis falsos para difamação e manipulações emocionais, como *gaslighting*.

*Eu namorei dois anos com ele, e essas ameaças aconteciam no relacionamento, principalmente quando eu queria terminar* [...]. *E, depois que eu terminei, pra ver se eu voltava e também para impedir que eu falasse alguma coisa que ele pudesse ter feito comigo, ou desabafasse com alguém pra não manchar a reputação dele.* (E25)

*Ele ainda tenta mandar* [solicitação de amizade nas redes sociais]. [...] *então, minhas redes sociais são todas privadas porque ele fez até fake para me controlar.* (E6)

A construção social do amor romântico influencia a percepção da ciberviolência, naturalizando-a e tornando os abusos difíceis de serem reconhecidos. Nesse sentido, relatos mostraram que o controle e a manipulação emocional iniciam sutilmente, disfarçados de atos de cuidado, evidenciando a romantização da possessividade.

*E quando você, que está sofrendo as coisas, eu acho que vai acontecendo tão aos poucos que você não vai se dando conta. Você não percebe. É só mais um dia, mais uma atitude, ele gosta tanto de mim. Ele está se preocupando comigo. E também as formas de manipulação acabam se tornando tão profundas que você fica mais apaixonada ainda.* (E21)

*Ele* [...] *começou a falar que não conseguia viver sem mim, que não conseguia ficar longe de mim por muito tempo. Daí é aquela de que no começo a gente acha fofo. A gente acha assim: “Nossa, ele não consegue viver sem mim, que dó”.* (E1)

A categoria “Sentimentos em relação à ciberviolência” abrange raiva, vergonha, medo e desvalorização pessoal. As falas destacaram a vergonha como um sentimento que acarretou o silenciamento de situações de violência por receio de o sofrimento ser considerado insignificante ou desacreditado. Já o sentimento de medo esteve relacionado à possibilidade de exposição pública das imagens íntimas e às consequências sociais e emocionais da ciberviolência.

*Eu não contei pra ninguém, porque você fica com vergonha, sabe? (*E29)

[...] *mesmo que eu esteja muito longe do local e nunca mais tenha visto ele, faz seis anos. Mesmo assim, eu ainda tenho medo.* (E25)

O sentimento de desconforto foi evidenciado no relato de um dos participantes, que afirmou ter sido pressionado por sua namorada a participar de atividades sexuais *online*, tendo cedido à pressão por acreditar que, por ser homem, não deveria demonstrar vulnerabilidade. Ademais, informou que cedeu por medo de consequências negativas e pela percepção de que uma denúncia da violência sofrida por um homem não seria levada a sério.

*Eu acabava deixando acontecer* [atos sexuais], *porque eu tinha medo de que pudesse ter alguma repercussão ruim se eu não fizesse. Ou porque eu pensava que sendo o cara da relação, não devia ser eu que devia me sentir desconfortável.* (E18)

A categoria “Consequências da ciberviolência” revelou os impactos dessa forma de violência na saúde e no cotidiano das vítimas. Os relatos mostraram sentimentos de invalidação e culpa que acarretavam questionamentos sobre seu próprio valor e a legitimidade de suas reações. Destaca-se que uma das entrevistadas afirmou se sentir forçada a se mudar para outro estado, enfrentando consequências como isolamento, preconceito e agravamento do seu estado emocional.

*Por muito tempo, mexeu com a minha autoestima.* [...] *eles* [amigos do ex-namorado] *falaram coisas da minha aparência, que eu era louca, enfim. Eu acho que mexeu por muito tempo, porque eu comecei a colocar na minha cabeça que talvez eles estivessem certos pelas coisas que eu implicava* [...]. (E9)

*Eu tive que ir embora.* [...] *acho que as consequências me acompanham até hoje* [...] *e o tanto que eu fiquei deprimida por estar aqui longe de onde eu morava e de onde eu era.* [...] *aqui eu sofri várias questões raciais e étnicas. Acho que foi um efeito dominó desde a ciberviolência até eu chegar aqui.* (E25)

Os participantes apontaram que pessoas em situação de ciberviolência podem enfrentar crises de pânico, ansiedade, depressão e, em alguns casos, tentar contra a própria vida em decorrência do sofrimento emocional causado pela ciberviolência.

*Eu comecei a ter crise de pânico. Eu não sabia o que era crise de pânico.* [...] *e num dia, eu tive uma crise muito forte de pânico, e aí eu fui internada, porque todo dia eu tinha e eu achava que eu ia morrer.* (E25)

[...] *já teve caso de uma amiga minha ficar se sentindo tão humilhada, tão deprimida, que ela tentou tirar a própria vida porque a família descobriu.* (E6)

Os participantes declararam que o isolamento, a falta de concentração e o desgaste emocional em virtude da ciberviolência frequentemente acarretam consequências negativas para a vida acadêmica, como faltas às aulas, reprovações e atrasos no cronograma acadêmico. Isso se deve à fragilidade do estado psicológico, que prejudica o desempenho universitário, gerando dificuldades em acompanhar as aulas e realizar provas.

*Logo quando ele fez o que ele tinha dito* [divulgação de imagens íntimas sem consentimento], *eu me isolei bastante.* [...] *então, eu deixava de ir à escola.* [...] *eu deixava de ir, eu atrasei um ano. E foi bem difícil assim pra eu voltar pra ela* [escola] *de novo.* (E25)

*Eu fui mal numa prova, eu faltei uma aula e depois, quando começaram as ameaças e ele começou a me perseguir, eu, às vezes, ficava nervosa quando eu estava na faculdade, mais distraída do que o normal*. (E6)

## DISCUSSÃO

As formas de ciberviolência entre parceiros íntimos mais vivenciadas pelos participantes foram ameaças e controle, seguidas pela exposição não consensual de informações íntimas e IBSA, geralmente acompanhadas de manipulação e coerção. Entre os casos mencionados sobre terceiros, o IBSA foi o mais citado, podendo ser reflexo da ideia do senso comum de que a ciberviolência entre parceiros íntimos se resume à divulgação de imagens íntimas.

Contudo, essa percepção simplificada não abrange a complexidade das vivências de quem sofre esse tipo de violência. Comportamentos como controle excessivo, ameaças, manipulação emocional e *gaslighting* são recorrentes em relações abusivas, mas, por vezes, permanecem invisíveis até culminar em episódios explícitos, como o IBSA^(14)^. Em geral, essas experiências não são compartilhadas com a rede de apoio, sendo reconhecidas apenas quando se concretizam em exposições públicas ou violência presencial. Assim, infere-se que a ciberviolência entre parceiros íntimos abrange um conjunto amplo e invisibilizado de práticas abusivas que extrapola os casos de exposição. Além disso, a ciberviolência pode ser o primeiro indício de abuso em relacionamentos íntimos e se manifestar articulada a outras modalidades de violência, aspecto já evidenciado em estudos anteriores^(15,16)^.

A categoria “Percepções sobre a ciberviolência” revela que os participantes percebem a violência no ambiente digital como um meio de punição ou vingança utilizado por homens contra mulheres, explorando sua desvantagem social nesse espaço. As experiências de mulheres que enfrentam ciberviolência diferem das vivências masculinas, pois estão marcadas pelas desigualdades de gênero, reforçando a necessidade de analisar essas questões sob uma perspectiva que considere essa desigualdade^(11)^.

A ciberviolência como violência de gênero foi identificada em pesquisa realizada com jovens árabes, a qual constatou sua manifestação por meio da coerção e das desigualdades de poder entre homens e mulheres em uma sociedade patriarcal. Nessa, os homens são incentivados a mostrar agressividade, adotar comportamentos controladores e promover discriminação de gênero com base em estereótipos. Enquanto isso, as mulheres são ensinadas a aceitar e se submeter aos homens, expondo-as à exploração e ao abuso, no mundo real e no ambiente virtual^(5)^.

Outro exemplo da ciberviolência como violência de gênero foi detectado em estudo que investigou discursos e práticas de violência contra meninas e mulheres em fóruns anônimos na internet: os *chans*. O estudo analisou 9,5 milhões de posts em dez *chans* e constatou que, em 46% das discussões sobre mulheres nesses fóruns, foram empregados termos violentos para se referir a elas, percentual que sobe para 69% quando as conversas estão relacionadas à pornografia. Nesses espaços, as mulheres são vistas como alvo de ressentimento, ódio e objetificação^(17)^.

Embora a internet pareça um espaço de neutralização das diferenças de gênero, observa-se que ela reproduz e intensifica estereótipos generificados presentes na sociedade. Assim, a ciberviolência emerge como um instrumento de reafirmação da masculinidade hegemônica, transpondo para o ambiente digital as mesmas assimetrias de poder observadas no contexto *offline* ^(5,18)^.

Os resultados do estudo em tela indicam que a ciberviolência se manifesta precocemente na vida de mulheres e é frequentemente naturalizada, tornando rara a experiência de chegar à vida adulta sem vivências de abuso. Por isso, uma análise centrada exclusivamente na perspectiva da vulnerabilidade de gênero não seria abrangente o suficiente para compreender o fenômeno, sendo necessário analisar também pelo prisma geracional.

Alguns estudos associam o IBSA à imprudência ou impulsividade dos adolescentes, reforçando a culpabilização das vítimas^(19,20)^. Outros, cujo enfoque é o desenvolvimento neurológico, estabelecem associação entre o envio de mídias eróticas e a falta de consciência ou o subdesenvolvimento do córtex pré-frontal em adolescentes^(15,21)^. Contudo, essas abordagens ignoram que tais práticas também ocorrem de forma planejada entre adultos, desconsiderando a complexidade estrutural da ciberviolência^(16)^.

A ideia de imprudência também pode ser constatada nas narrativas das participantes que vivenciaram a ciberviolência quando culpam a si mesmas pelas violências sofridas, interpretando essas experiências como resultado de comportamentos considerados inadequados, ingênuos ou impulsivos, tidos como próprios da adolescência. Dessa forma, reproduzem a lógica da culpabilização das vítimas, perpetuando os estigmas que as cercam^(22,23)^.

Portanto, a análise dos resultados deste estudo, sob a ótica geracional como parte da interpretação da realidade objetiva pela TIPESC, evidencia que a vulnerabilidade dos jovens à ciberviolência é acentuada pela inserção crescente e cada vez mais precoce no ambiente digital como local para início e manutenção de relacionamentos afetivos e sexuais^(3,22)^. Nesse contexto, o enfrentamento da ciberviolência exige a inclusão das interações digitais nas estratégias de prevenção, devido ao seu impacto na vida de meninas e mulheres.

A categoria “Vivência da ciberviolência” aponta essa forma de violência ser comum em relacionamentos amorosos, sendo experienciada principalmente por meio de ameaças e controle. Corroborando esses resultados, estudo qualitativo revelou que o controle é um denominador comum nas experiências de ciberviolência por parceiro íntimo e que, mesmo após o rompimento, houve aumento no contato indesejado com o ex-parceiro, que tentava manter influência sobre a mulher. Para isso, os abusadores utilizaram diversas tecnologias, como rastreamento de localização e hackeamento de contas, resultando em invasões de privacidade, ligações constantes e mensagens ameaçadoras^(14)^.

A naturalização dos comportamentos controladores está alicerçada no discurso patriarcal de que são demonstrações de amor e cuidado. Essa construção social que associa amor romântico à posse desempenha um papel central na percepção da ciberviolência, pois dificulta o reconhecimento de comportamentos abusivos e legitima atitudes abusivas e a vigilância digital dentro do relacionamento. Com isso, reforçam a dependência emocional e perpetuam o ciclo de violência^(3)^. Superar essas dinâmicas requer uma transformação social capaz de questionar e ressignificar o amor romântico, desafiando as normas culturais que vinculam controle e possessividade ao cuidado.

A categoria “Sentimentos em relação à ciberviolência” aponta as emoções negativas desencadeadas pela vivência da ciberviolência, com destaque para a vergonha. Esse sentimento frequentemente leva as vítimas a silenciar e evitar buscar ajuda, temendo que suas experiências sejam minimizadas, especialmente em contextos de relacionamento.

A vergonha, comum em situações de violência contra mulheres, intensifica-se em casos de exposição não autorizada da intimidade, funcionando como um mecanismo que perpetua a violência e dificulta a denúncia. Salienta-se que a vergonha é resultado de um processo de internalização no qual a vítima adota avaliações negativas sobre si mesma. Culturalmente, a honra está associada aos homens, enquanto a vergonha é atribuída às mulheres, reforçando uma hierarquia que desvaloriza a imagem feminina^(24)^.

Nesse sentido, a vergonha pode manter indivíduos em situações de violência, como evidenciado na fala de um entrevistado do sexo masculino que, apesar do desconforto, relatou ter participado de atividades sexuais *online* por pressão de sua namorada. Essa experiência pode ser analisada a partir da perspectiva patriarcal, que tem a virilidade como um ideal socialmente construído, representando simultaneamente um privilégio e uma armadilha. Assim, a virilidade - associada à força e à capacidade reprodutiva - impõe aos homens a obrigação constante de provar sua masculinidade, tornando-os vulneráveis a situações de opressão^(25,26)^. Nesse contexto, a pressão para corresponder às expectativas de gênero pode dificultar o reconhecimento e a denúncia de experiências abusivas vividas por homens, perdurando dinâmicas de violência pouco visibilizadas.

A categoria “Consequências da ciberviolência” evidencia que os impactos dessa forma de violência extrapolam o espaço *online* e afetam a vida cotidiana das vítimas, incluindo autoestima, confiança e interações sociais. Pessoas em situação de ciberviolência frequentemente enfrentam sentimentos de invalidação, culpa e medo, o que pode resultar em mudanças significativas na rotina, como evitar determinados locais ou até mudar de cidade^(27)^.

Esses impactos sociais são corroborados por autores que utilizam a expressão “morte social” para descrever os relatos de destruição moral perante a família, amigos e desconhecidos na internet vivida por pessoas em situação de ciberviolência. Ademais, há uma sensação de perseguição e insegurança que faz a pessoa se sentir constantemente vigiada e julgada, independentemente de onde esteja, mesmo que ninguém no local tenha acesso ao conteúdo violado^(27)^.

Os danos da ciberviolência são sentidos em níveis individuais e sistêmicos, afetando a privacidade, autonomia e bem-estar psicológico dos indivíduos^(11)^. Relativo à saúde mental, a ciberviolência pode desencadear depressão, crises de pânico, ansiedade e, em casos mais graves, tentativas de suicídio. Estudo realizado com 970 adolescentes de minorias sexuais, com idades entre 14 e 17 anos, investigou a relação entre o IBSA e comportamentos suicidas e de autolesão, constatando que a ameaça de divulgar esse tipo de conteúdo foi associada a um risco aumentado de ideação suicida, planejamento e tentativa de suicídio, além de autolesão. Já a postagem não consensual de mídias foi relacionada a um maior risco de ideação e tentativa de suicídio^(28)^.

Apesar de inúmeras pesquisas apontarem os impactos negativos da ciberviolência na saúde^(2,5,9,11,19,28)^, o setor da saúde ainda não a reconhece como um objeto de sua intervenção. Além disso, destaca-se a carência de estudos brasileiros sobre essa temática, o que pode estar relacionado à baixa visibilidade do fenômeno no país e consequente ausência de medidas eficazes para sua prevenção e enfrentamento.

Por isso, é necessário que as universidades, enquanto espaços privilegiados de promoção da saúde, incorporem o enfrentamento das violências, inclusive em suas manifestações digitais, como eixo central de suas políticas institucionais. Experiências como as “Diretrizes gerais para as ações institucionais de intervenção diante de situações de violência e discriminação de gênero e orientação sexual” da Universidade de São Paulo ilustram iniciativas relevantes ao estabelecer parâmetros normativos, serviços de apoio e protocolos de atenção à violência de gênero, reafirmando o compromisso das instituições de ensino superior com a construção de ambientes acadêmicos seguros, respeitosos e alinhados aos Objetivos do Desenvolvimento Sustentável^(29)^.

Destaca-se que a inclusão exclusiva de estudantes da área da saúde como participantes do estudo favorece a reflexão crítica sobre desigualdades e estruturas que perpetuam a violência. Tal processo pode qualificar a prática do cuidado no futuro, ao promover o reconhecimento das profissionais como agentes de mudança capazes de superar limitações impostas por processos históricos e culturais de dependência e subordinação, possibilitando a construção de práticas fundamentadas na equidade, nos direitos, na cidadania e na justiça social^(23)^.

### Limitações do estudo

Destaca-se como principal limitação deste estudo a baixa participação de homens, mesmo diante de convites frequentes. A resistência dos homens em abordar a violência, seja como vítimas ou agressores, contribuiu para que a pesquisa refletisse majoritariamente experiências femininas. Contudo, gera questionamentos sobre o silenciamento masculino em torno da temática da VPI, inclusive sobre a invisibilidade das violências que também vivenciam nas reações de intimidade.

### Contribuições para a área da enfermagem, saúde ou políticas públicas

O presente estudo analisa a ciberviolência sob uma perspectiva crítica, reconhecendo-a como um fenômeno emergente e ainda incipientemente abordado nos campos da saúde e da enfermagem. Trata-se de expressão contemporânea da violência de gênero, estruturada por assimetrias de poder derivadas das construções socioculturais de gênero e geração. Ao evidenciar seus impactos no processo de saúde-doença de jovens, sobretudo mulheres, esta investigação amplia a compreensão das dinâmicas simbólicas e relacionais que sustentam práticas de controle e dominação mediadas por tecnologias digitais.

Diante da escassez de estudos nacionais que explorem essa problemática sob uma ótica interseccional, os achados deste trabalho reafirmam o papel da enfermagem como uma prática ética, crítica e transformadora. A pesquisa contribui para a consolidação da ciberviolência como objeto de atenção nos campos da vigilância em saúde, da educação permanente e do cuidado interprofissional, sobretudo em espaços universitários. Além disso, oferece subsídios para o desenvolvimento de estratégias intersetoriais e intervenções comprometidas com a promoção da saúde, equidade, e construção de ambientes seguros, acolhedores e inclusivos.

## CONSIDERAÇÕES FINAIS

Os achados deste estudo evidenciam que a ciberviolência é uma realidade frequente entre jovens universitários, sobretudo mulheres, com início ainda na adolescência e maior incidência em contextos de relacionamentos íntimos, especialmente durante os rompimentos. Suas manifestações, como controle, coerção, assédio, exposição de imagens íntimas e perseguição, produzem impactos significativos na saúde física e mental, nas relações sociais e no desempenho acadêmico.

A ciberviolência se revela como expressão de uma violência simbólica sustentada por desigualdades estruturais de gênero e geração, as quais reforçam práticas que humilham, desumanizam e vulnerabilizam principalmente mulheres jovens. Ressalta-se que o referencial teórico-metodológico adotado neste estudo reconhece que uma perspectiva sensível às outras categorias sociais, como classe social e raça/etnia, é essencial para repensar as relações de poder nas dinâmicas de ciberviolência. Nos campos da saúde coletiva e da enfermagem, essa abordagem amplia a compreensão dos impactos da ciberviolência no processo de saúde-doença, associando-os às desigualdades estruturais.

Portanto, a superação da ciberviolência exige a transformação das relações de poder que a sustentam, sendo mais importante do que respostas punitivas. Sendo assim, este estudo contribui para esse debate, incentivando novas pesquisas e intervenções no contexto universitário que promovam equidade, respeito e justiça social.

## Data Availability

Os dados de pesquisa estão disponíveis no corpo do artigo.
